# An optimized protocol for assessment of sputum macrorheology in health and muco-obstructive lung disease

**DOI:** 10.3389/fphys.2022.912049

**Published:** 2022-08-05

**Authors:** Mirjam Völler, Annalisa Addante, Hanna Rulff, Benjamin von Lospichl, Simon Y. Gräber, Julia Duerr, Daniel Lauster, Rainer Haag, Michael Gradzielski, Marcus A. Mall

**Affiliations:** ^1^ Department of Pediatric Respiratory Medicine, Immunology and Critical Care Medicine, Charité—Universitätsmedizin Berlin, Corporate Member of Freie Universität Berlin and Humboldt-Universität zu Berlin, Berlin, Germany; ^2^ German Centre for Lung Research (DZL), Associated Partner Site, Berlin, Germany; ^3^ Institute of Chemistry, Technische Universität Berlin, Berlin, Germany; ^4^ Berlin Institute of Health at Charité—Universitätsmedizin Berlin, Berlin, Germany; ^5^ Institute of Chemistry and Biochemistry, Freie Universität Berlin, Berlin, Germany

**Keywords:** mucus, sputum, bovine submaxillary mucin, macrorheology, solvent trap, cystic fibrosis, muco-obstructive lung disease

## Abstract

**Background:** Airway mucus provides important protective functions in health and abnormal viscoelasticity is a hallmark of muco-obstructive lung diseases such as cystic fibrosis (CF). However, previous studies of sputum macrorheology from healthy individuals and patients with CF using different experimental protocols yielded in part discrepant results and data on a systematic assessment across measurement settings and conditions remain limited.

**Objectives:** The aim of this study was to develop an optimized and reliable protocol for standardized macrorheological measurements of airway mucus model systems and native human sputum from healthy individuals and patients with muco-obstructive lung disease.

**Methods:** Oscillatory rheological shear measurements were performed using bovine submaxillary mucin (BSM) at different concentrations (2% and 10% solids) and sputum samples from healthy controls (*n* = 10) and patients with CF (*n* = 10). Viscoelastic properties were determined by amplitude and frequency sweeps at 25°C and 37°C with or without solvent trap using a cone-plate geometry.

**Results:** Under saturated atmosphere, we did not observe any temperature-dependent differences in 2% and 10% BSM macrorheology, whereas in the absence of evaporation control 10% BSM demonstrated a significantly higher viscoelasticity at 37°C. Similarly, during the measurements without evaporation control at 37°C we observed a substantial increase in the storage modulus G′ and the loss modulus G″ of the highly viscoelastic CF sputum but not in the healthy sputum.

**Conclusion:** Our data show systematically higher viscoelasticity of CF compared to healthy sputum at 25°C and 37°C. For measurements at the higher temperature using a solvent trap to prevent evaporation is essential for macrorheological analysis of mucus model systems and native human sputum. Another interesting finding is that the viscoelastic properties are not much sensitive to the applied experimental deformation and yield robust results despite their delicate consistency. The optimized protocol resulting from this work will facilitate standardized quantitative assessment of abnormalities in viscoelastic properties of airway mucus and response to muco-active therapies in patients with CF and other muco-obstructive lung diseases.

## Introduction

In recent years, there has been a renewed interest in studying the macrorheology of airway mucus in health and muco-obstructive lung diseases such as cystic fibrosis (CF). Physiologically, airway mucus keeps airway surfaces hydrated and entraps inhaled toxins, particles, allergens and pathogens that are constantly cleared from the lungs by mucociliary clearance (MCC) (Knowles and Boucher, 2002; Mall, 2008; Hill et al., 2022). Normal airway mucus consists of ∼97% water and ∼3% dispersed material (mucins, other proteins, lipids, salt and cellular debris, e.g., DNA) (Fahy and Dickey, 2011). Mucins are strongly-glycosylated macromolecules with high water-binding capacity (Hattrup and Gendler, 2008; Thornton et al., 2008; Ridley and Thornton, 2018). By disulfide, ionic, and noncovalent cross-linking, mucin monomers can form gel-like multimeric networks with closely regulated biophysical properties between a viscous fluid and an elastic solid (Thornton et al., 2008; Lai et al., 2009; Fahy and Dickey, 2011; Meldrum et al., 2018; Trillo-Muyo et al., 2018; Morrison et al., 2019). Altered sputum viscoelasticity has been demonstrated in a spectrum of muco-obstructive lung diseases including CF, non-CF bronchiectasis, asthma, and chronic obstructive pulmonary disease (COPD) (Dunican et al., 2018; Lin et al., 2020; Ramsey et al., 2020). CF is one of the most common lethal autosomal recessive disorders caused by mutations in the cystic fibrosis membrane conductance regulator (CFTR) gene leading to impaired function of the CFTR anion channel that plays a key role in the secretion of chloride, bicarbonate and fluid across airway epithelia (Ehre et al., 2014; Mall and Hartl, 2014; Sanders and Fink, 2016; Mall et al., 2020). CFTR dysfunction results in mucus hyperconcentration, disturbed mucin expansion upon exocytosis, increased mucin entanglement and cross-linking (Button et al., 2012; Anderson et al., 2015; Yuan et al., 2015; Hill et al., 2018; Morrison et al., 2019), leading to highly viscoelastic sputum and impaired MCC (Hoegger et al., 2014; Anderson et al., 2015). The resulting clinical hallmarks are mucus plugging, impaired ventilation, chronic airway inflammation and infection, and progressive lung damage (Liou et al., 2001; Zhou-Suckow et al., 2017; Wine et al., 2018; Balazs and Mall, 2019; Esther et al., 2019; Bell et al., 2020; Turcios, 2020).

Previous studies reported that the degree of altered sputum macrorheology correlates with other outcome measures of disease severity including lung function, sputum microbiome and inflammation markers (Tomaiuolo et al., 2014; Ma et al., 2018; Patarin et al., 2020). These data suggest sputum macrorheology as a promising biomarker for monitoring disease severity and progression, as well as response to therapy. However, the interpretation of previous studies of sputum macrorheology is hampered as the protocols and thus the obtained ranges of sputum viscoelasticity in health and disease differ substantially (Shah et al., 1996; Dwyer et al., 2008; Innes et al., 2009; Lai et al., 2009; Yuan et al., 2015; Vukosavljevic et al., 2017; Zhou-Suckow et al., 2017; Ridley and Thornton, 2018; Ghanem et al., 2020). For example, there are discrepancies in relation to the measurement temperature and whether a solvent trap was used to control for evaporation and mucus concentration during the measurement (Dwyer et al., 2008; Innes et al., 2009; Lai et al., 2009; Yuan et al., 2015; Vukosavljevic et al., 2017; Zhou-Suckow et al., 2017; Ghanem et al., 2020; Patarin et al., 2020). A systematic assessment of potential advantages and disadvantages of measuring sputum macrorheology under different conditions has not been performed (Knowles and Boucher, 2002; Lai et al., 2009; Ghanem et al., 2020; Patarin et al., 2020).

The aim of this study was therefore to develop an optimized and robust protocol for the assessment of airway mucus macrorheology in health and disease by systematically characterizing bulk rheology in a bovine submaxillary mucin (BSM) model system and native human sputum. BSM was chosen as mucus model system for protocol design and optimization, as it is commercially available and easy to prepare in quantities sufficient to perform the measurements under different experimental conditions in the same sample. In order to assess the influence of mucin concentration, measurement temperature, and evaporation control we first studied BSM macrorheology at different concentrations (2% and 10% solids to mimic healthy and CF mucus, respectively) and measurement conditions (25°C versus 37°C, with versus without a solvent trap) by dynamic oscillatory shear measurements consisting of an amplitude- and a strain-controlled frequency sweep using a cone-plate geometry. Next, we examined the macrorheology of native sputum samples from healthy controls and patients with CF under the same experimental conditions for optimized measurement of sputum macrorheology as a potential biomarker for CF and possibly other muco-obstructive pulmonary diseases.

## Material and methods

### Study design and participants

This observational study was approved by the ethics committee of the Charité—Universitätsmedizin Berlin (EA2/016/18). Written informed consent to participate in this study was provided by all participants, parents or legal guardians. Exclusion criteria were history of smoking, acute respiratory infection or pulmonary exacerbation, a history of organ transplantation, or prior exposure to CFTR modulator treatment. Healthy controls had no history of any chronic lung disease. Demographics and clinical characteristics of study participants [healthy individuals (*n* = 10) and CF patients (*n* = 10)] are shown in Supplementary Table S1.

### Sputum collection

In healthy controls and CF patients, sputum was induced by inhalation of hypertonic saline (NaCl 6%) using a PARIBOY system in combination with PARI LC PLUS nebuliser (PARI GmbH, Starnberg, Germany). Sputum was collected after expectoration, saliva was removed by gentle aspiration with a pipette, and sputum samples were immediately put on ice and directly subjected to macrorheology measurements.

### Preparation of mucus from bovine submaxillary mucin

BSM (Mucin, Bovine Submaxillary Gland, Merck KGaA, Darmstadt, Germany) was dissolved in Dulbecco’s phosphate buffered saline (DPBS) buffer without calcium or magnesium (Gibco, Thermo Fisher Scientific, Waltham, MA, United States) for 45 min at room temperature with a magnetic stirrer at the lowest speed to prevent the formation of bubbles. We prepared 2% and 10% solutions to mimic the concentrations of healthy and CF sputum, respectively (Henderson et al., 2014; Boucher, 2019; Hill et al., 2022). The samples were measured directly after preparation.

### Macrorheology

Macrorheological measurements were performed immediately after BSM preparation or native sputum collection with a Kinexus Pro+ Rheometer (NETZSCH GmbH, Selb, Germany). All experiments were performed using a stainless-steel cone-plate geometry (cone-diameter 20 mm, cone-angle 1°). Native sputum samples were separated from saliva and possible debris and transferred onto the lower static plate of the rheometer with a non-electrostatic spatula. After confining the sample with the upper rotating cone, the non-electrostatic spatula was also used to trim any excess sample. We conducted dynamic oscillatory measurements to characterize the linear macrorheological properties of the samples. To test whether these properties were independent of strain deformation, we performed frequency sweeps in the range of 0.1–10 Hz at five different deformation amplitudes (0.5%, 1%, 2%, 5%, and 10%) using 10% BSM and studied the obtained curves.

To test the impact of temperature and evaporation, measurements were repeated with a new aliquot of the same sample under four different experimental conditions. Specifically, measurements were performed with and without a passive solvent trap (NETZSCH GmbH, Selb, Germany) first at 25°C and afterwards at 37°C. To ensure the atmosphere in this closed environment was saturated at any time during the experiment, a defined amount of 900 μl distilled water was added into the upper reservoir of the solvent trap. After loading and between the performed sequences, the samples were equilibrated for 5 minutes at the given temperature to ensure full temperature equilibration and sufficient network relaxation. Each sequence included an amplitude sweep and a frequency sweep down- and upwards. The amplitude sweep was performed at a fixed frequency of 1 Hz and covered a range of shear deformation *γ* between 0.01%–10%. The frequency sweep was conducted at a fixed shear deformation of 2% and covered a frequency range of 0.05–50 Hz. An entire measurement sequence lasted approximately 45 min.

In a first step we determined the linear viscoelastic (LVE) region within the amplitude sweeps, which represents the quasi-static behavior of the material commonly at lower strains. Second, the frequency sweeps were analyzed regarding the behavior of the storage modulus G′ and loss modulus G″. The material properties were quantified by the phase angle *δ* which is defined as:

$$\tan\delta =\frac{G^{\prime \prime }}{G^{\prime }}$$

A phase angle *δ* = 0° indicates perfect elastic deformation behavior, a phase angle *δ* = 90° perfect viscous flow behavior. If *δ* < 45°, the material is dominated by its elastic properties; if *δ* > 45°, the material is dominated by its viscous properties.

To estimate an effective mesh size *ξ* of the mucin network, we used the following formula (Rehner, 1943; Hoffmann, 1994):

$$\xi =\;{\left(\frac{k_{B}\cdot T}{G}\right)}^{\frac{1}{3}}$$

with k_B_ being the Boltzmann constant, T the absolute temperature and G the shear modulus, which we approximated by the G′ value at 1 Hz.

### Statistical analysis

Data were analyzed using GraphPad Prism version 9.0.1 (GraphPad Software, San Diego, CA, United States) and are reported as mean ± standard error of the mean (SEM). A Kruskal–Wallis test followed by Dunn’s multiple comparison was applied to test for statistical significance. *p* < 0.05 was accepted to indicate statistical significance.

## Results

### Initial experiments to determine the effect of strain amplitude in the bovine submaxillary mucin mucus model system

**FIGURE 1:**
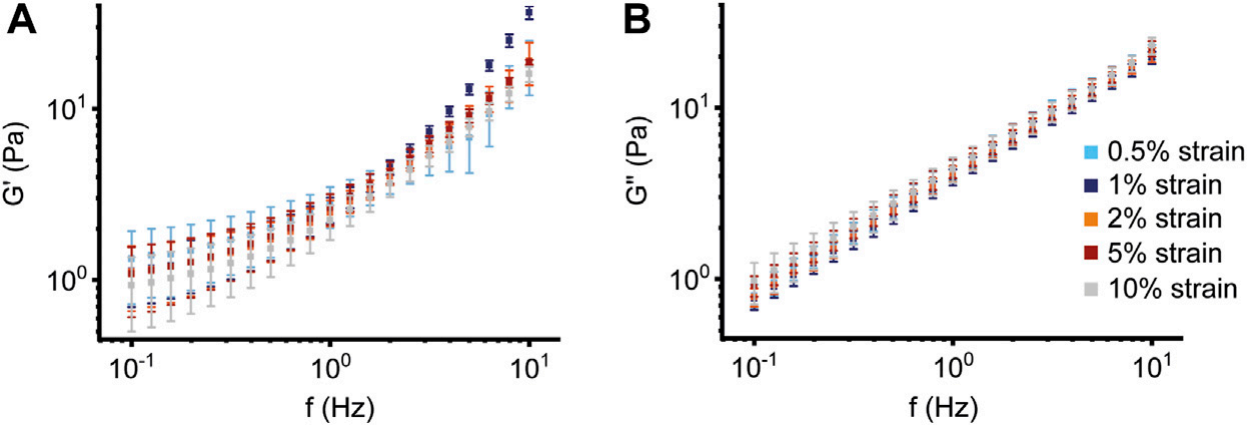
Deformation effect of different strain amplitudes. Effect of different strain amplitudes (0.5%–1%–2%–5%–10%) as function of frequency (Hz) on **(A)** storage modulus G′ and **(B)** loss modulus G″ of 10% bovine submaxillary mucin (*n* = 5) measured at 37°C with solvent trap.

To identify conditions that enable standardized and stable measurements of the macrorheological properties of highly viscoelastic CF sputum, we used BSM as a mucus model with a “CF-like” concentration of 10% solids. First, to determine if the measured linear macrorheological properties are independent of the deformation, we performed several oscillatory shear test experiments applying different strain amplitudes from 0.5% to 10% in a frequency range of 0.1–10 Hz and analyzed the storage modulus G′ and the loss modulus G″ (Figure 1; Supplementary Figure S1). We observed that the values for G′ and G″ of 10% BSM showed very similar behavior within the investigated frequency range when comparing the values for different applied strains. The moduli were not affected by the choice of the strain amplitude in this range. The phase angle > 45° indicated that 10% BSM tends to behave more like a viscoelastic fluid (Supplementary Table S2). In addition, we observed continuously increasing moduli across the investigated frequency range indicating that none of the applied deformations up to strain amplitudes of 10% modified the linear macrorheological properties of the investigated mucus samples in the experimental frequency range (Figure 1). These data show that the five strain amplitudes tested in these experiments did not compromise the linear rheological properties of 10% BSM. Based on these results, all subsequent rheological measurements using frequency sweeps were performed with a strain amplitude of 2%, thereby being safely in a fully reliable amplitude range.

### Effect of mucin concentration, temperature and evaporation control on macrorheology of the bovine submaxillary mucin mucus model system

**FIGURE 2:**
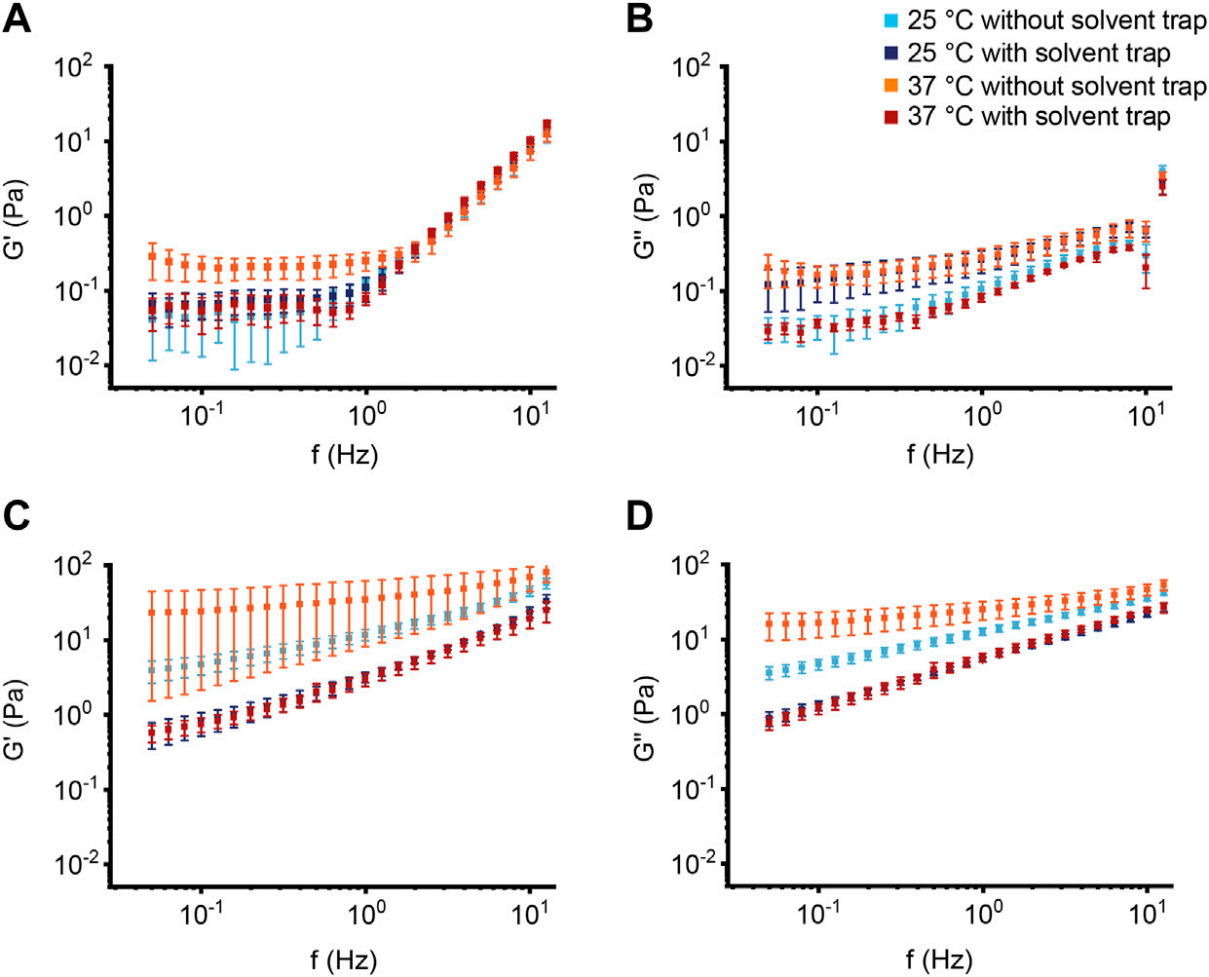
Effect of mucin concentration, measurement temperature and evaporation control on macrorheology of the bovine submaxillary mucin mucus model system. **(A,C)** Storage modulus G′ and **(B,D)** loss modulus G″ of **(A,B)** 2% (*n* = 5) and **(C,D)** 10% (*n* = 5) bovine submaxillary mucin were measured as function of frequency (Hz). Data are shown as mean ± standard error of the mean (SEM) of measurements at 25°C and 37°C with and without solvent trap.

**FIGURE 3:**
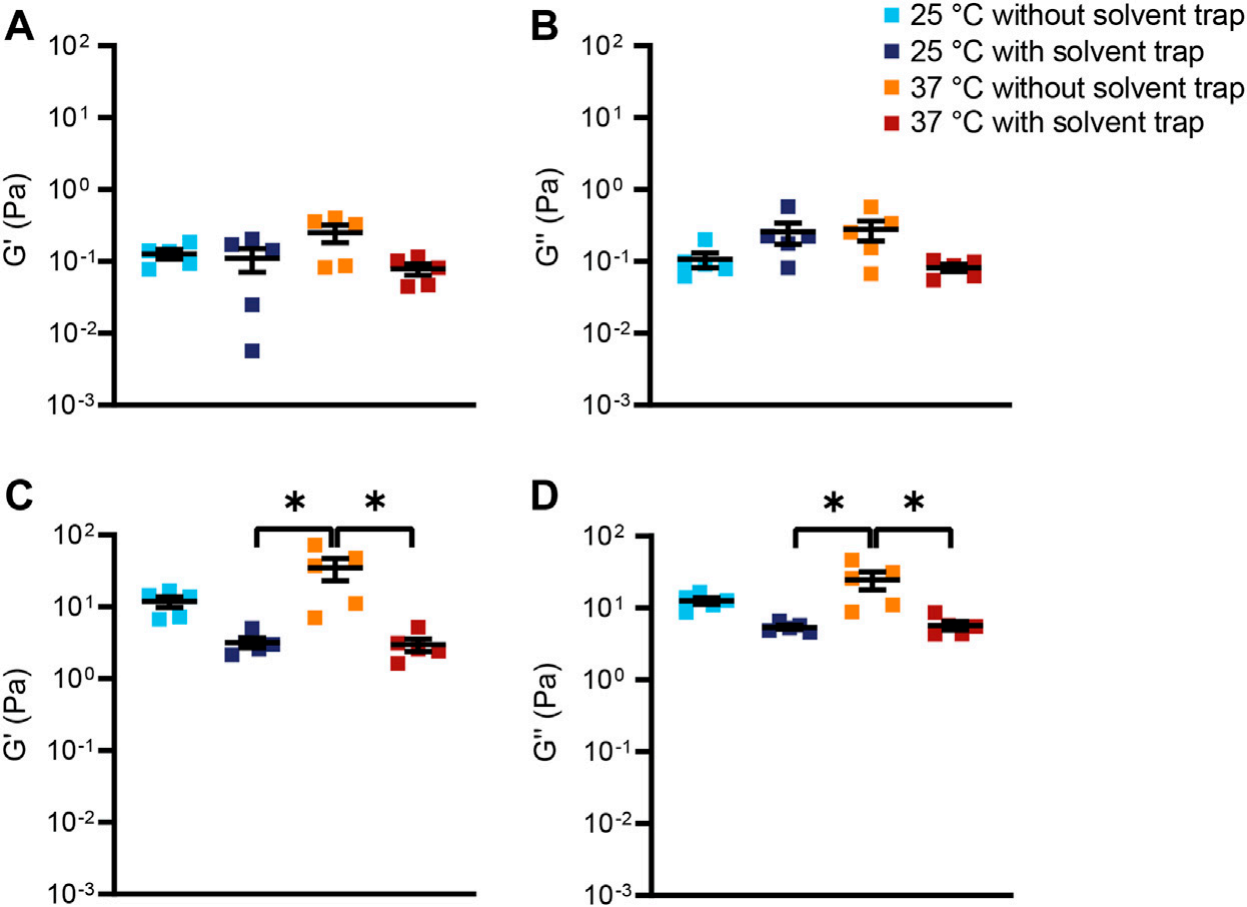
Viscoelastic properties of bovine submaxillary mucin. **(A,C)** Storage modulus G′ and **(B,D)** loss modulus G″ of **(A,B)** 2% (*n* = 5) and **(C,D)** 10% (*n* = 5) bovine submaxillary mucin were measured at a frequency of 1 Hz. Data are shown as mean ± standard error of the mean (SEM) of measurements at 25°C and 37°C with and without solvent trap; *p < 0.05.

To systematically evaluate the effects of mucus concentration, temperature and evaporation on macrorheology of our mucus model system, we repeated oscillatory measurements using 2% and 10% BSM to mimic concentrations of healthy and CF sputum at 25°C and 37°C, both with and without solvent trap, respectively. 25°C was chosen since it is the most commonly used standard measurement temperature, while 37°C is the physiological body temperature. Both the storage and loss modulus of 2% BSM remained relatively stable up to a frequency of 1 Hz and increased at higher frequencies. In 10% BSM, both moduli continuously increased over the entire frequency range. 10% BSM showed nearly two orders of magnitude higher values for G′ and G″ compared to 2% BSM showing that the elastic properties and thus the resulting estimated effective mesh size of BSM are strongly concentration-dependent (Figures 2, 3; Supplementary Table S2). Further, we observed that the elastic properties tended to become more dominant at higher BSM concentration, indicated by a decreasing computed phase angle *δ* in 10% compared to 2% BSM (Supplementary Figure S2; Supplementary Table S2). For 2% BSM, there was no difference in G′ between 25°C and 37°C under saturated atmosphere using a solvent trap, but a trend toward a higher G′ at 37°C without solvent trap compared to measurements with solvent trap (Figures 2A, 3A; Supplementary Table S2). Similarly, G″ of 2% BSM did not differ between 25°C and 37°C with solvent trap and showed a trend towards higher G″ at 37°C without solvent trap (Figures 2B, 3B; Supplementary Table S2). Also for 10% BSM we did not observe any substantial differences between G′ and G″ measured at the two different temperatures in the presence of the solvent trap (Figures 2, 3; Supplementary Table S2). However, in contrast to 2% BSM, 10% BSM showed significantly higher values for G′ and G″ at 37°C without solvent trap compared to measurements performed at 37°C and 25°C with solvent trap (Figures 2C,D, 3C,D; Supplementary Table S2). Moreover, the results were more variable at 37°C under non-saturated atmosphere, indicating that evaporation during the oscillatory experiment that lasted around 45 min, rendered the experiments erroneous and poorly reproducible at the higher temperature in the absence of a solvent trap.

### Effect of temperature and evaporation control on macrorheology of healthy and cystic fibrosis sputum

**FIGURE 4:**
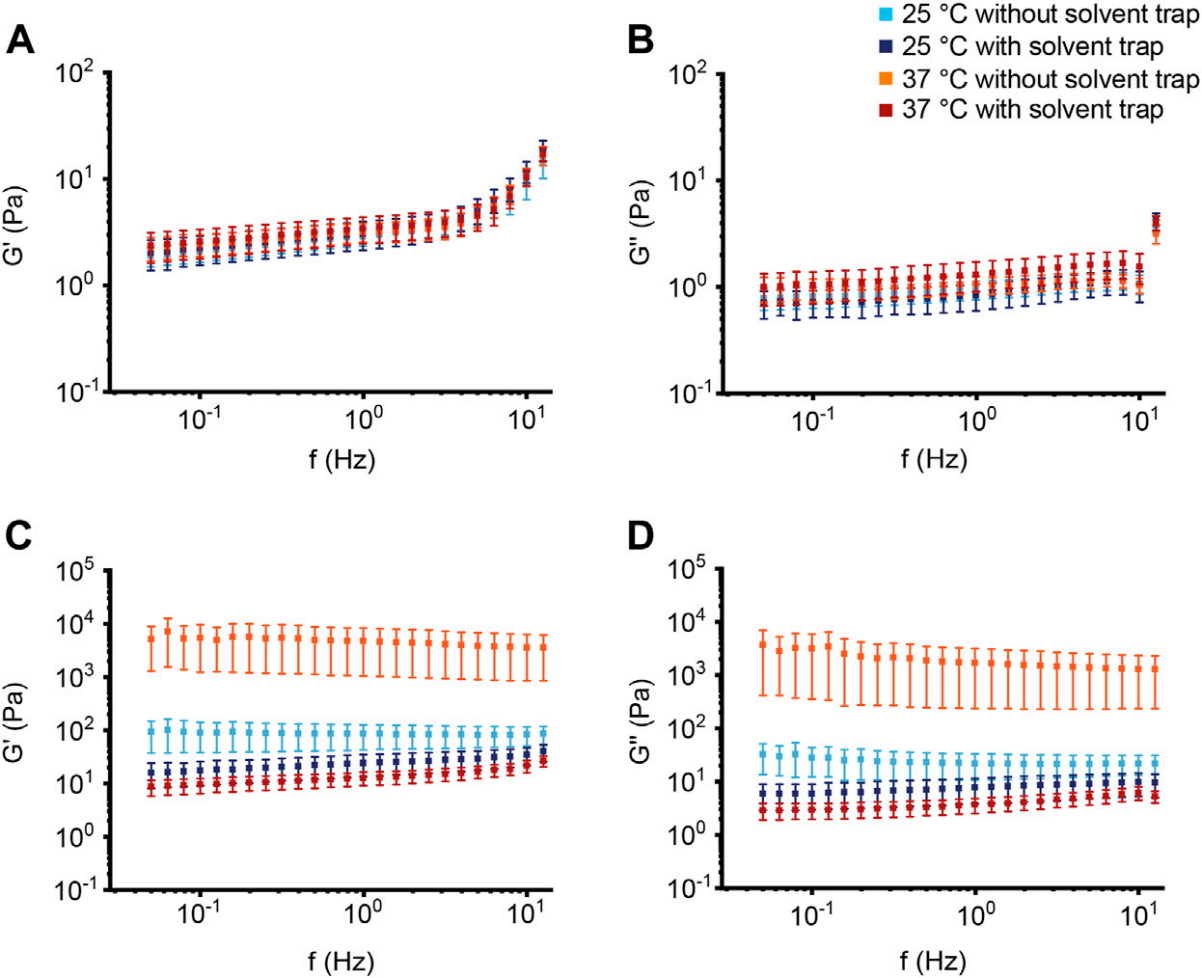
Effect of temperature and saturated atmosphere on macrorheology of healthy and cystic fibrosis sputum. **(A,C)** Storage modulus G′ and **(B,D)** loss modulus G″ of sputum from **(A,B)** healthy controls (*n* = 10) and from **(C,D)** patients with CF (*n* = 10) were measured as function of frequency (Hz). Data are shown as mean ± standard error of the mean (SEM) of measurements at 25°C and 37°C with and without solvent trap.

**FIGURE 5:**
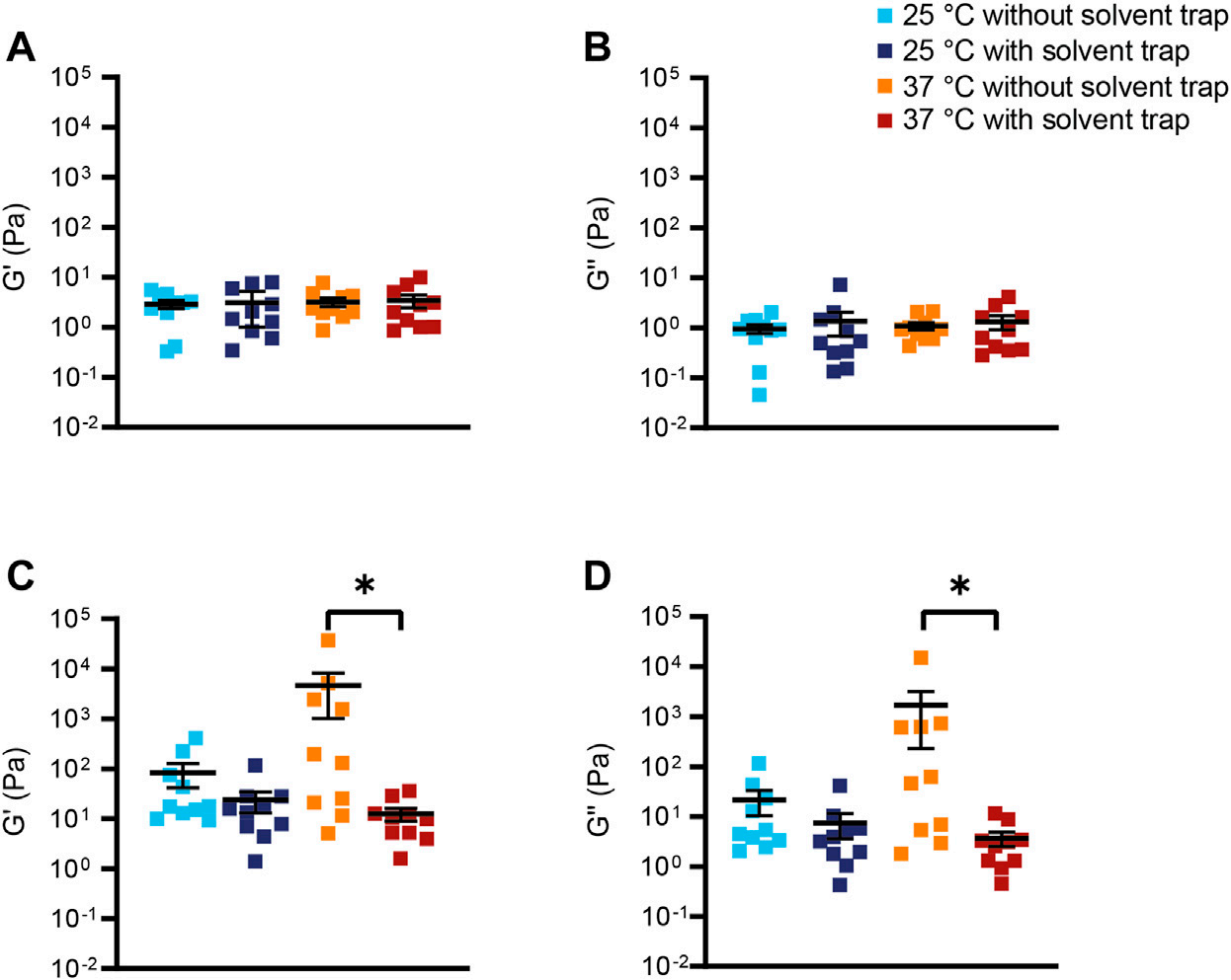
Viscoelastic properties of human sputum. **(A,C)** Storage modulus G′ and **(B,D)** loss modulus G″ of **(A,B)** sputum from healthy controls (*n* = 10) and from **(C,D)** patients with cystic fibrosis (*n* = 10) were measured at a frequency of 1 Hz. Data are shown as mean ± standard error of the mean (SEM) of measurements at 25°C and 37°C with and without solvent trap; *p < 0.05.

**FIGURE 6:**
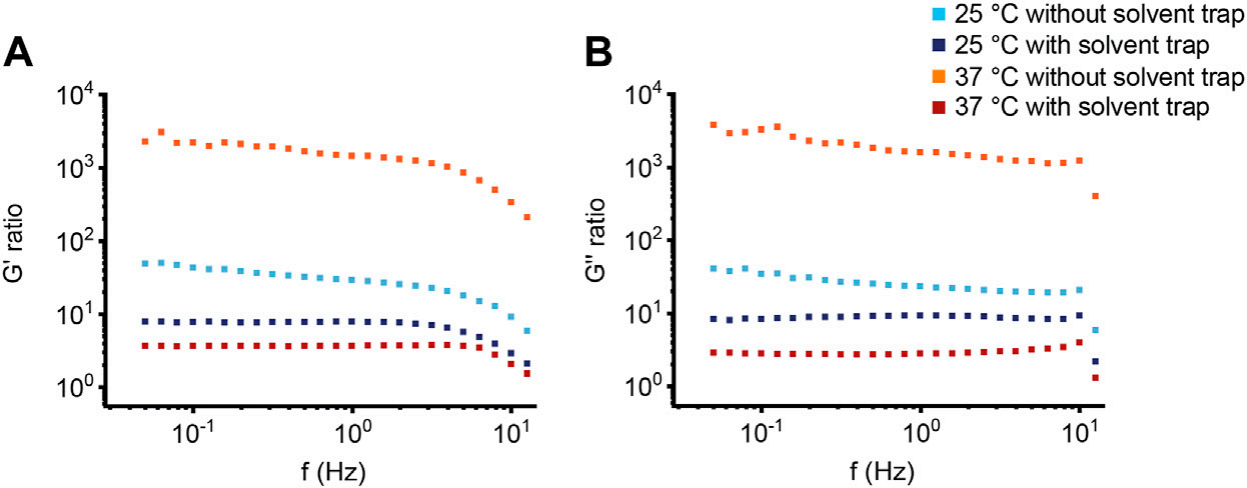
Differences in sputum viscoelasticity between healthy individuals and patients in cystic fibrosis. Ratio of **(A)** the storage modulus G′ and **(B)** the loss modulus G″ between sputum from patients with cystic fibrosis (*n* = 10) and sputum from healthy controls (*n* = 10) of measurements at 25°C and 37°C with and without solvent trap.

Next, to evaluate whether our observations from the BSM can be translated into the more complex human mucus system, the macrorheological properties of sputum from healthy subjects and patients with CF were characterized (Supplementary Table S1). Whereas the storage modulus G′ increased at higher frequencies, the loss modulus G″ of healthy sputum was not impacted by frequency over the entire measurement range from 0.05 to 50 Hz (Figures 4A,B). The CF samples showed stable values for G′ and G'' in the measured frequency range (Figures 4C,D). As expected from previous studies (Dawson et al., 2003; Patarin et al., 2020; Lai et al.,2009), sputum from patients with CF showed substantially higher values for G′ and G″ across all measurement conditions resulting in smaller estimated effective sputum mesh sizes (Figures 4–6; Supplementary Figure S3; Supplementary Table S3). In healthy as well as CF sputum, G′ predominated G″, i.e., human sputum behaved like a viscoelastic solid when probed by macrorheological techniques. This was also supported by a phase angle of ∼19° in healthy and ∼16° in CF sputum, respectively (Supplementary Figure S2; Supplementary Table S3). As observed for 2% BSM, G′ and G″ of sputum from healthy subjects behaved similar across all four experimental setups (Figures 4A,B, 5A,B; Supplementary Table S3). In contrast to our findings in healthy sputum, in the absence of the solvent trap, G′ and G″ of CF sputum at 37°C were markedly increased compared to G′ and G″ with solvent trap (Figures 4C,D, 5C,D; Supplementary Table S3). In addition, at 37°C without solvent trap, we observed a discrepancy between the viscoelastic moduli from the downwards and the subsequently performed upwards frequency sweeps (increasing G′ with time by about a factor of three for the highest frequency), indicating an increasing viscoelasticity of the CF sputum samples in the course of the measurement, likely caused by evaporation due to the non-saturated atmosphere during the entire measurement sequence (Supplementary Figure S4).

## Discussion

In this study, we investigated the impact of mucus concentration, temperature and humidity/evaporation control on macrorheological properties of BSM as a mucus model system, and native sputum from healthy subjects and patients with CF as a prototypical muco-obstructive lung disease in order to define an optimized macrorheological protocol. Our results show that deformations up to 10% strain amplitude do not interfere with the linear macrorheological parameters in the CF-like 10% BSM mucus model system (Figure 1), despite the fact that mucus constitutes a delicate hydrogel. Further, we demonstrate that BSM at different concentrations can be used to model viscoelastic differences between native healthy sputum and pathological sputum from patients with CF (Figures 2, 4). Under saturated atmosphere, we did not observe any temperature-dependent differences in 2% and 10% BSM, whereas in the absence of evaporation control 10% BSM demonstrated a significantly higher viscoelasticity at 37°C (Figures 2, 3). Similarly, we observed a substantial increase in G′ and G″ of the highly viscoelastic CF sputum during the measurements without evaporation control, especially at higher temperature (Figures 4–6; Supplementary Figure S4).

To our knowledge, this is the first study directly comparing measurements with and without solvent trap at different measurement temperatures using both BSM as a mucus model system and human sputum samples. The general goal of a solvent trap is to control the local humidity around the sample by preventing evaporation. As expected, it has been demonstrated that evaporation is a key mechanism influencing the water content of the mucus layer (Karamaoun et al., 2018). Some of the previously published macrorheology protocols already included a solvent trap to prevent evaporation (Dawson et al., 2003; Serisier et al., 2009; Tomaiuolo et al., 2014). However, there is still a relevant fraction of macrorheology studies where the use of a solvent trap has not been described (Shah et al., 1996; Dwyer et al., 2008; Innes et al., 2009; Yuan et al., 2015; Vukosavljevic et al., 2017; Patarin et al., 2020). Consistent with previous studies in different hydrogel systems and studies using native human samples, we did not observe any significant temperature-dependent effects comparing measurements at room temperature versus 37°C under evaporation control (Tomaiuolo et al., 2014; Lafforgue et al., 2018). This confirms that viscoelasticity arises primarily from permanent crosslinks and not from chain entanglements. Further, our results confirm physicochemical properties previously reported for healthy and CF sputum analyzed under saturated atmosphere, including viscoelasticity (range of G′ and G″) and derived estimated effective mesh pore size reported by other groups using a solvent trap (Figures 4, 5; Supplementary Table S3) (Sanders et al., 2000; Dawson et al., 2003). In addition, our data demonstrate that using the solvent trap is essential to prevent evaporation and dehydration of the sample during the course of the measurement, especially when carried out at the physiological temperature of 37°C (Figures 2–6, Supplementary Tables S2, S3). In the absence of evaporation control, the network structure became stiffer during the course of the measurement, which was particularly relevant for highly viscoelastic samples (10% BSM and CF sputum) (Figures 2–5, Supplementary Figure S4). Collectively, these results highlight the importance of humidity control for the assessment of changes in viscoelastic properties of sputum from patients with muco-obstructive lung diseases such as CF.

Our measurements in the BSM mucus model are consistent with previous studies demonstrating that mucin concentration plays an important role in determining the macrorheology of mucus hydrogels (Figures 2, 3; Supplementary Table S2) (Henderson et al., 2014; Kesimer et al., 2017; Sharma et al., 2021). In line with previous studies, our measurements in human sputum showed that both healthy and CF sputum were dominated by the elastic component (G′ > G″, phase angle < 20°) characteristic of solid- or gel-like behavior (Figures 4, 5; Supplementary Figure S2; Supplementary Table S3) (Dawson et al., 2003; Tomaiuolo et al., 2014; Patarin et al., 2020). This predominance of G′ together with a similar frequency-dependence of both moduli is characteristic of cross-linked polymers (Dawson et al., 2003; Tomaiuolo et al., 2014). In contrast, although BSM demonstrated an increasing dominance of the elastic properties with higher concentration, the phase angle of 10% BSM was still not within the range of native sputum (Supplementary Figure S2; Supplementary Tables S2, S3). We speculate that this difference in viscoelastic properties of BSM compared with human sputum may reflect differences in the extent of cross-linking, or impurities of the BSM including bovine serum albumin that may contribute to a higher viscosity (Nikogeorgos et al., 2014). In addition, native human sputum contains biomolecules missing in the purified BSM samples that could account for enhanced elastic properties. While these differences in viscoelastic properties have to be kept in mind, our results support BSM as a useful and easily accessible tool that may be applicable for preclinical testing, e.g., of therapeutic strategies designed to improve mucus macrorheology to establish a proof-of-concept before testing limited patient samples.

The standardized protocol for assessment of mucus macrorheology established in our study may be combined with micro-rheological approaches, as well as analyses of mesoscopic structure to provide an in-depth characterization of the biophysical and structural properties of airway mucus in health in order to better understand the desired state of mucus, as well as the effects of pH, salt concentration, osmolytes and interaction with viral and bacterial pathogens on mucus structure and function in health (Lai et al., 2009; Madsen et al., 2014; Schuster et al., 2014; Hill et al., 2018; Hill et al., 2022). In addition, such comparative studies from the “macro” to the “micro” scale of different mucus sources, including mucus model systems, mucus from primary airway epithelial cells or patient sputum, are expected to increase our current knowledge on the underlying causes of altered viscoelastic properties of airway mucus in CF and other muco-obstructive lung diseases and may thus provide a basis for future diagnostic and therapeutic approaches (App et al., 1998). In this context, several studies showed that sputum viscoelasticity correlated with the severity of airway infection and inflammation, as well as lung function impairment in CF patients indicating that sputum macrorheology may be a promising biomarker for monitoring of disease severity (Tomaiuolo et al., 2014; Ma et al., 2018). However, larger studies including a robust and reproducible macrorheology protocol are needed to test its potential as biomarker of disease activity in the clinical arena. In these future studies, it would also be interesting to address effects of proteases on mucus processing and viscoelastic properties in health and disease (Innes et al., 2009; Dittrich et al., 2018; McKelvey et al., 2020; Rickert-Zacharias et al., 2021). Proteases can be released from airway inflammatory cells, bacteria and epithelial cells, and previous studies of sputum from patients with CF indicated that host- and pathogen-derived proteases, such as human neutrophil elastase and *P. aeruginosa* elastase B, can degrade airway mucins *in vivo* (Henke et al., 2011). Because the activity of these proteolytic enzymes is temperature-dependent and temperature varies as a function of airway region (i.e., ∼32°C in the upper airways versus ∼35°C in the bronchial regions), local differences in airway temperature may influence protease-mediated mucus processing differently along the tracheobronchial tree. However, the relationship between local temperature, proteolytic processing and viscoelastic properties of airway mucus remains poorly understood. Our protocol could be utilized to address this question by adding pathophysiological relevant proteases, as well as protease inhibitors to sputum from healthy controls and patients with muco-obstructive lung diseases, and by including additional temperatures in the measurements. In addition, our optimized protocol may be used to monitor the effect of therapeutic strategies, such as mucolytic therapies targeting disulfide crosslinks with reducing agents, or novel CFTR-directed therapeutics targeting the underlying basic defect in patients with CF (Shak et al., 1990; Rubin, 2006; Henke and Ratjen, 2007; Lai et al., 2009; Mall et al., 2018; Ehre et al., 2019; Fernandez-Petty et al., 2019; Middleton et al., 2019; Patarin et al., 2020; Morgan et al., 2021).

This study also has limitations. Due to the small volume of native sputum samples available, all measurements were performed using a small cone-plate geometry (cone-diameter 20 mm), precluding assessment of potential effects of a larger cone-plate geometry (and consequently a larger sample volume) on the results. Moreover, the sample size of CF patients in this cross-sectional study is too small to determine associations of the rheological parameters with disease severity. Therefore, larger longitudinal studies will be required to establish the relationship between rheological outcomes and quantitative outcome measures of airway inflammation, infection and lung function that will be important to establish sputum macrorheology assessed with this protocol as a biomarker of disease severity and response to therapy in patients with CF and potentially other muco-obstructive lung diseases.

In summary, our study provides a systematic analysis of the influence of temperature and humidity control on rheological properties of sputum from healthy subjects and patients with CF. We demonstrate that the presence of a solvent trap to avoid evaporation is indispensable for performing measurements at physiological temperature to keep the macrorheological behavior of the sample constant during the measurement. Humidity control was especially important for assessment of samples with high mucin concentration such as CF sputum. On the other side we also demonstrate that the choice of the deformation in the experiment is not really crucial for the outcome, at least if one stays below values of 10% for the deformation. Our data support that this optimized protocol can be used to assess sputum macrorheology in health and in muco-obstructive lung diseases, where it may serve as a potential biomarker of disease severity and response to therapy.

## Data Availability

The raw data supporting the conclusion of this article will be made available by the authors, without undue reservation.
