# Supplementary material for: An optimized protocol for assessment of sputum macrorheology in health and muco-obstructive lung disease
Source: Front Physiol. 2022 Aug 5;13:912049. doi: 10.3389/fphys.2022.912049 (PMC9388721; doi:10.3389/fphys.2022.912049)
Supplement: Supplementary file 1 [file DataSheet1.pdf]

Supplementary Material

Supplementary Figures and Tables

Table S1. Demographics and basic clinical characteristics of healthy controls and patients with cystic fibrosis.

	Healthy controls Mean (± SEM) or n (%)	Patients with cystic fibrosis Mean (± SEM) or n (%)
Number of samples	10	10
Age, years	33.6 (± 1.6)	35.2 (± 4.9)
Sex, female	8 (80%)	8 (80%)
Pancreatic insufficiency	n.d.	6 (60%)
BMI, kg/m²	26.0 (± 1.4)	21.2 (± 1.4)
FEV_1 % predicted	n.d.	58.5 (± 4.6)

Abbreviations: BMI = body mass index; FEV_1 = forced expiratory flow in one second; SEM = standard error of the mean; n.d. = not determined.

Table S2. Summary of the macrorheological parameters of 2% and 10% bovine submaxillary mucin.

		2% BSM		10% BSM	
		**mean ± SEM**	***P* value**	**mean ± SEM**	***P* value**
**G' (Pa) at 1 Hz**	25 °C without solvent trap	0.1 ± 0.0		11.8 ± 2.0	
	25 °C with solvent trap	0.1 ± 0.0		3.2 ± 0.5	*
	37 °C without solvent trap	0.3 ± 0.1		35.1 ± 12.0	
	37 °C with solvent trap	0.1 ± 0.0		3.0 ± 0.6	*
**G'' (Pa) at 1 Hz**	25 °C without solvent trap	0.1 ± 0.02		12.6 ± 1.3	
	25 °C with solvent trap	0.3 ± 0.1		5.4 ± 0.4	*
	37 °C without solvent trap	0.3 ± 0.1		24.8 ± 7.0	
	37 °C with solvent trap	0.1 ± 0.0		5.7 ± 0.8	*
**Mesh size (nm) at 1 Hz**	25 °C without solvent trap	326.0 ± 17.0		72.6 ± 4.6	
	25 °C with solvent trap	463.1 ± 120.6		111.1 ± 5.1	
	37 °C without solvent trap	284.7 ± 34.7		58.0 ± 8.8	
	37 °C with solvent trap	392.9 ± 25.8		116.6 ± 7.2	
**Phase angle (°) at 1 Hz**	25 °C without solvent trap	72.3 ± 10.8		48.1 ± 2.8	
	25 °C with solvent trap	65.2 ± 11.0		60.0 ± 3.7	*
	37 °C without solvent trap	57.4 ± 9.2		39.5 ± 3.7	**
	37 °C with solvent trap	54.9 ± 10.8		63.2 ± 1.8	***

Mean values and standard error of the mean (SEM) of the storage modulus G’ (Pa), loss modulus G’’ (Pa), effective mesh size (nm) and phase angle (°) of 2% (n = 5) and 10% (n = 5) bovine submaxillary mucin (BSM) measured at a frequency of 1 Hz at 25 °C and 37 °C with and without solvent trap. **P*<0.05, ***P*<0.01, ****P*<0.001.

Table S3. Summary of the macrorheological parameters of sputum from healthy controls and patients with cystic fibrosis.

		Healthy sputum		Cystic Fibrosis sputum	
		**mean ± SEM**	***P* value**	**mean ± SEM**	***P* value**
**G' (Pa) at 1 Hz**	25 °C without solvent trap	2.9 ± 0.5		84.0 ± 42.2	
	25 °C with solvent trap	3.1 ± 0.9		23.9 ± 10.7	
	37 °C without solvent trap	3.4 ± 1.0		4634 ± 3612	] *
	37 °C with solvent trap	3.2 ± 0.6		12.5 ± 3.5	
**G'' (Pa) at 1 Hz**	25 °C without solvent trap	0.9 ± 0.2		21.7 ± 11.2	
	25 °C with solvent trap	1.4 ± 0.7		7.5 ± 3.9	
	37 °C without solvent trap	1.3 ± 0.4		1700 ± 1470	] *
	37 °C with solvent trap	1.1 ± 0.2		1.1 ± 0.2	
**Mesh size (nm) at 1 Hz**	25 °C without solvent trap	131.1 ± 15.9		53.8 ± 6.2	
	25 °C with solvent trap	136.8 ± 15.6		73.8 ± 9.7	
	37 °C without solvent trap	119.5 ± 8.0		38.0 ± 9.6	
	37 °C with solvent trap	125.3 ± 10.9		82.1 ± 8.3	
**Phase angle (°) at 1 Hz**	25 °C without solvent trap	17.4 ± 1.6		15.0 ± 0.7	
	25 °C with solvent trap	16.6 ± 1.1		15.9 ± 0.9	
	37 °C without solvent trap	21.0 ± 3.0		16.7 ± 1.3	
	37 °C with solvent trap	20.3 ± 1.7		15.5 ± 0.9	

Mean values and standard error of the mean (SEM) of the storage modulus G’ (Pa), loss modulus G’’ (Pa), effective mesh size (nm) and phase angle (°) of sputum from healthy controls (n = 10) and patients with cystic fibrosis (n = 10) measured at a frequency of 1 Hz at 25 °C and 37 °C with and without solvent trap. *$P<0.05$.

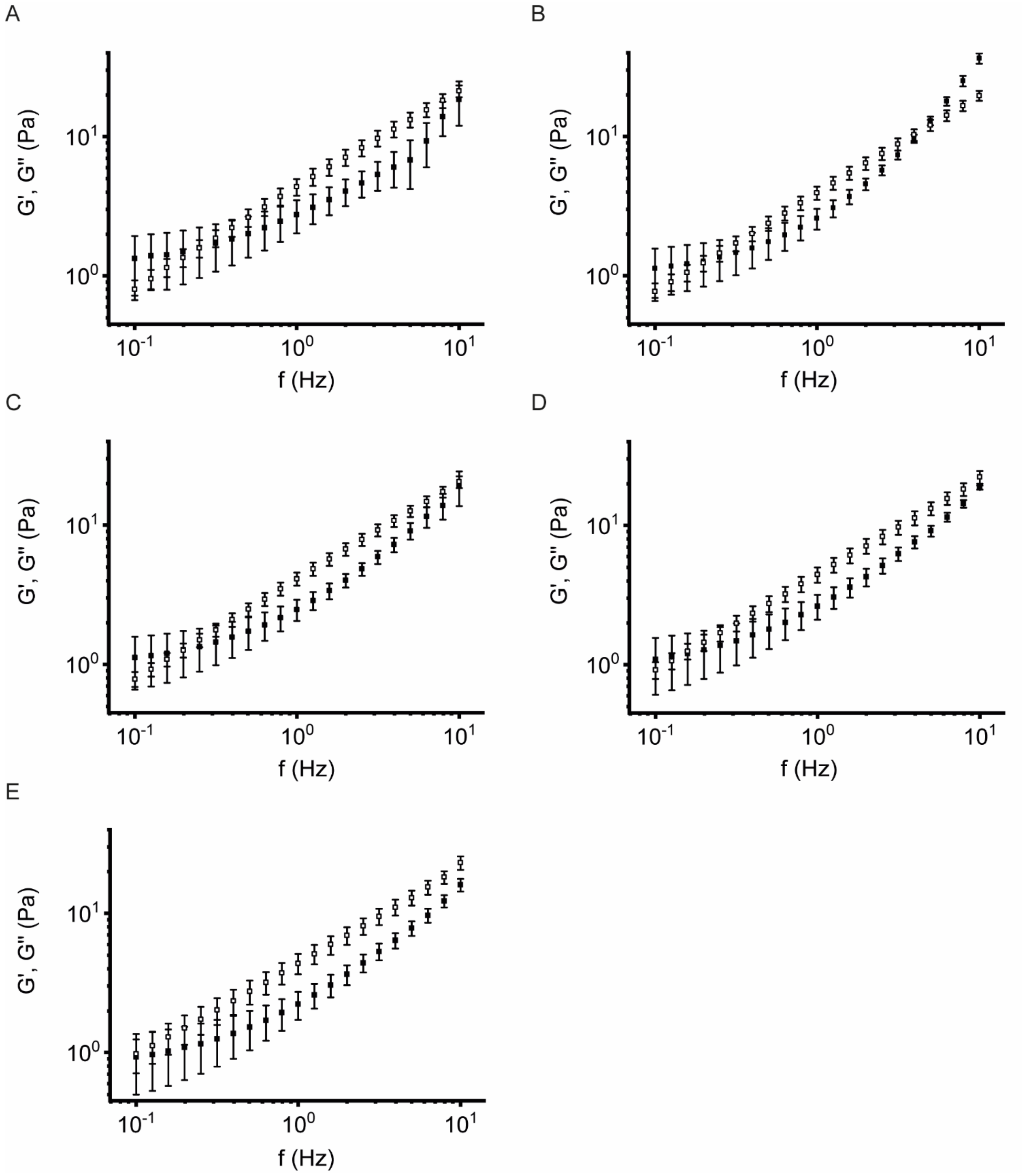

Figure S1. Deformation effect of different strain amplitudes. Storage modulus G' (closed squares) and loss modulus G'' (open squares) of different strain amplitudes 0.5% **(A)** - 1% **(B)** - 2% **(C)** - 5% **(D)** - 10% **(E)** as function of frequency (Hz) of 10% bovine submaxillary mucin (n = 5) measured at 37 °C with solvent trap.

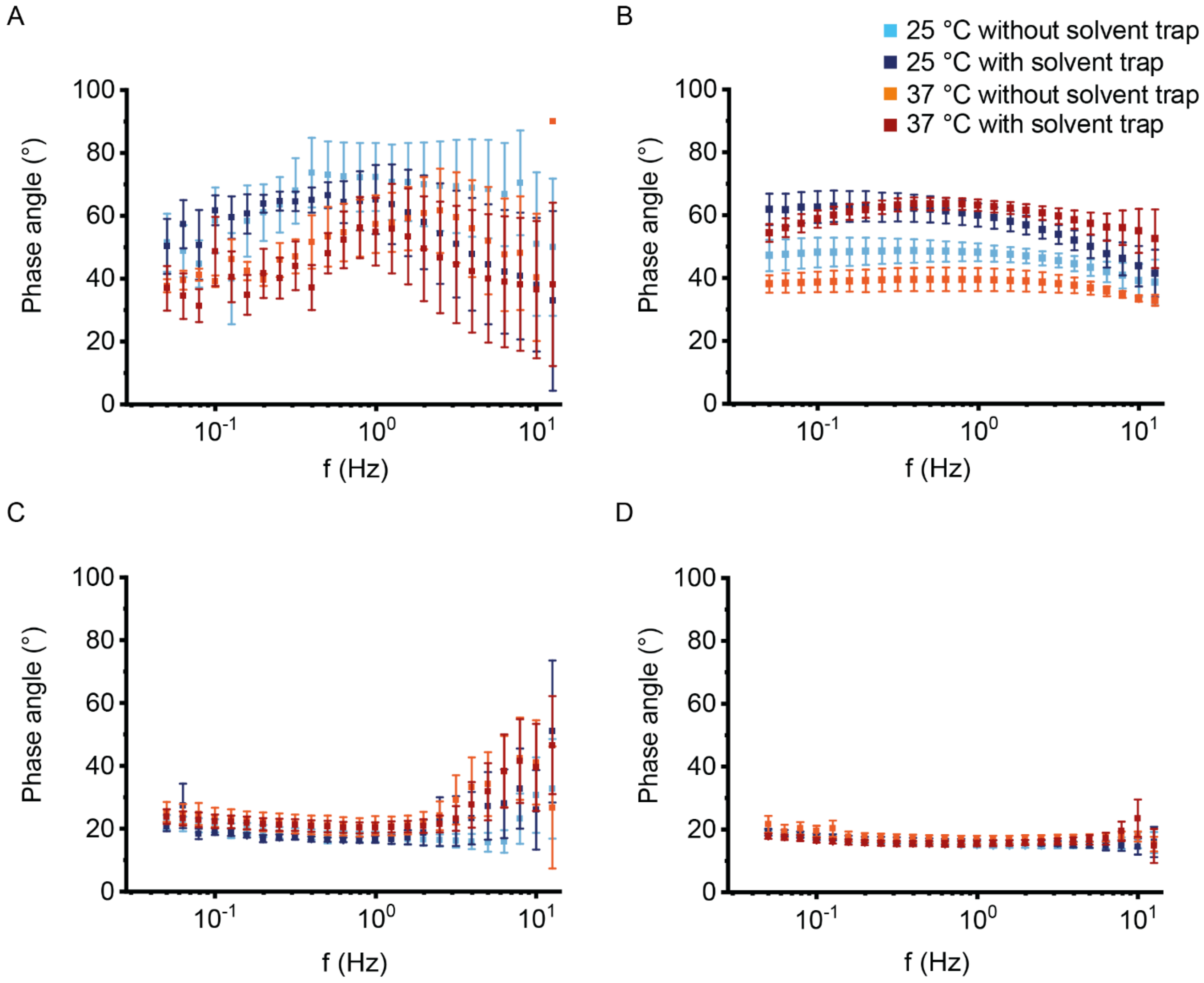

Figure S2. Phase angle of bovine submaxillary mucin and human sputum. Phase angle (°) or loss factor of **(A)** 2% (n = 5) and **(B)** 10% (n = 5) bovine submaxillary mucin and of **(C)** sputum from healthy controls (n = 10) and **(D)** patients with cystic fibrosis (n = 10) as function of frequency (Hz). Data are shown as mean ± standard error of the mean (SEM) of measurements at 25 °C and 37 °C with and without solvent trap.

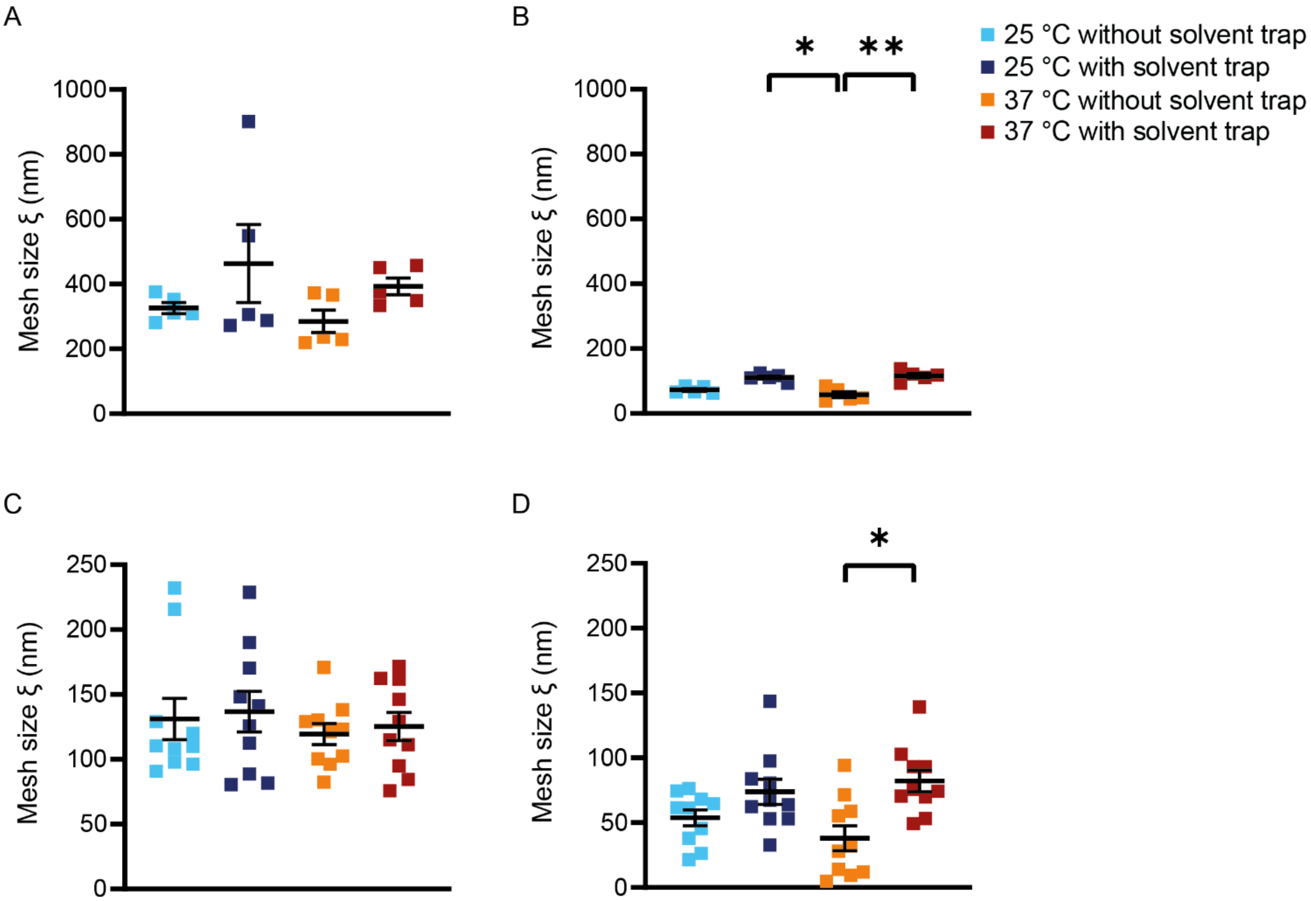

Figure S3. Mesh size of bovine submaxillary mucin and human sputum. Effective mesh size ξ (nm) of **(A)** 2% (n = 5) and **(B)** 10% (n = 5) bovine submaxillary mucin and of sputum from **(C)** healthy controls (n = 10) and **(D)** patients with cystic fibrosis (n = 10). Data are shown as mean ± standard error of the mean (SEM) of measurements at 25 °C and 37 °C with and without solvent trap; $^{*}P<0.05$, $^{**}P<0.01$.

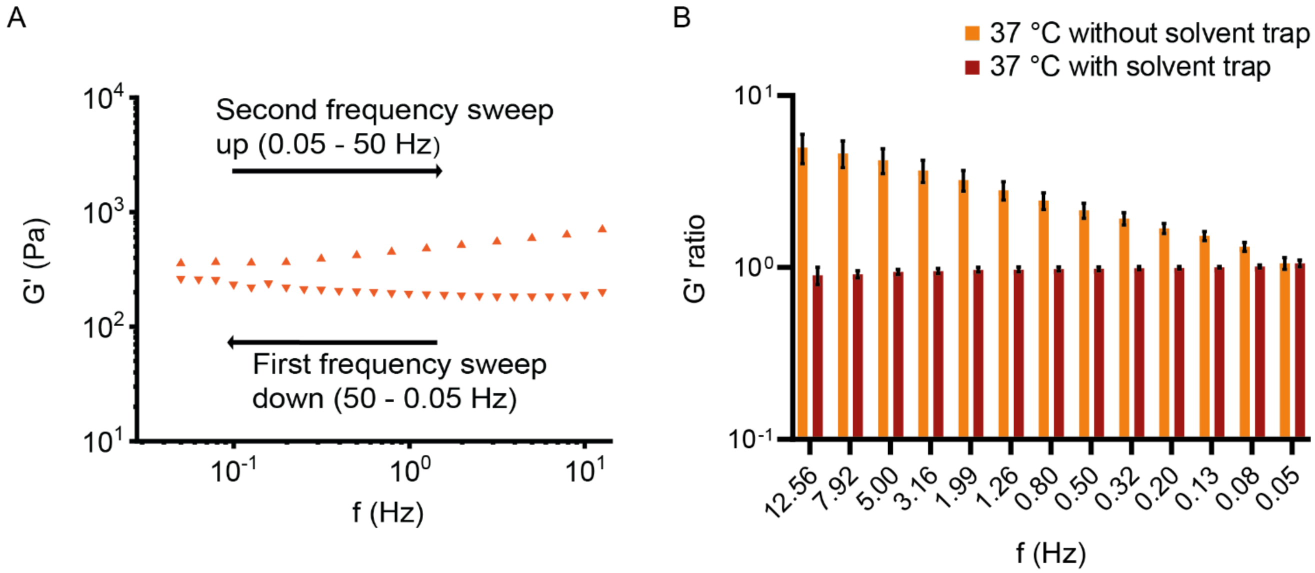

Figure S4. Hysteresis loop of patients with cystic fibrosis. (**A**) Representative frequency sweep downwards (down-pointing triangle) and upwards (up-pointing triangle) of sputum from a patient with cystic fibrosis at 37 °C without solvent trap. (**B**) Ratio between the storage modulus G' (Pa) of sputum from patients with cystic fibrosis (n = 10) between the frequency sweep upwards and the frequency sweep downwards at 37 °C without or with solvent trap, respectively. For each frequency mean and standard error of the mean (SEM) is depicted.
