# Supplementary material for: An optimized protocol for assessment of sputum macrorheology in health and muco-obstructive lung disease
Source: Front Physiol. 2022 Aug 5;13:912049. doi: 10.3389/fphys.2022.912049 (PMC9388721; doi:10.3389/fphys.2022.912049)
Supplement: Supplementary file 2 [file DataSheet1.docx]

Supplementary Material

## Supplementary Figures and Tables

| **Table S1. Demographics and basic clinical characteristics of healthy controls and patients with cystic fibrosis.** | | | | | |
| --- | --- | --- | --- | --- | --- |
|  |  |  |  |  |  |
|  | |  | **Healthy controls**  **Mean (± SEM)**  **or n (%)** |  | **Patients with cystic fibrosis**  **Mean (± SEM)**  **or n (%)** |
| Number of samples | |  | 10 |  | 10 |
| Age, years | |  | 33.6 (± 1.6) |  | 35.2 (± 4.9) |
| Sex, female | |  | 8 (80%) |  | 8 (80%) |
| Pancreatic insufficiency | |  | n.d. |  | 6 (60%) |
| BMI, kg/m² | |  | 26.0 (± 1.4) |  | 21.2 (± 1.4) |
| FEV_1_ % predicted | |  | n.d. |  | 58.5 (± 4.6) |

Abbreviations: BMI = body mass index; FEV_1_ = forced expiratory flow in one second; SEM = standard error of the mean; n.d. = not determined.

**Table S2. Summary of the macrorheological parameters of 2% and 10% bovine submaxillary mucin.**

|  |  | **2% BSM** | | **10% BSM** | |
| --- | --- | --- | --- | --- | --- |
|  |  | **mean ± SEM** | ***P* value** | **mean ± SEM** | ***P* value** |
| **G' (Pa)**  **at 1 Hz** | 25 °C without solvent trap | 0.1 ± 0.0 |  | 11.8 ± 2.0 | 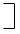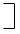  *  * |
|  | 25 °C with solvent trap | 0.1 ± 0.0 |  | 3.2 ± 0.5 |  |
|  | 37 °C without solvent trap | 0.3 ± 0.1 |  | 35.1 ± 12.0 |  |
|  | 37 °C with solvent trap | 0.1 ± 0.0 |  | 3.0 ± 0.6 |  |
| **G'' (Pa)**  **at 1 Hz** | 25 °C without solvent trap | 0.1 ± 0.02 |  | 12.6 ± 1.3 | 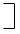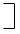  *  * |
|  | 25 °C with solvent trap | 0.3 ± 0.1 |  | 5.4 ± 0.4 |  |
|  | 37 °C without solvent trap | 0.3 ± 0.1 |  | 24.8 ± 7.0 |  |
|  | 37 °C with solvent trap | 0.1 ± 0.0 |  | 5.7 ± 0.8 |  |
| **Mesh size (nm) at 1 Hz** | 25 °C without solvent trap | 326.0 ± 17.0 |  | 72.6 ± 4.6 |  |
|  | 25 °C with solvent trap | 463.1 ± 120.6 |  | 111.1 ± 5.1 |  |
|  | 37 °C without solvent trap | 284.7 ± 34.7 |  | 58.0 ± 8.8 |  |
|  | 37 °C with solvent trap | 392.9 ± 25.8 |  | 116.6 ± 7.2 |  |
| **Phase angle (°) at 1 Hz** | 25 °C without solvent trap | 72.3 ± 10.8 |  | 48.1 ± 2.8 | 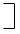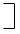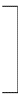  ***  *  ** |
|  | 25 °C with solvent trap | 65.2 ± 11.0 |  | 60.0 ± 3.7 |  |
|  | 37 °C without solvent trap | 57.4 ± 9.2 |  | 39.5 ± 3.7 |  |
|  | 37 °C with solvent trap | 54.9 ± 10.8 |  | 63.2 ± 1.8 |  |

Mean values and standard error of the mean (SEM) of the storage modulus G’ (Pa), loss modulus G’’ (Pa), effective mesh size (nm) and phase angle (°) of 2% (n = 5) and 10% (n = 5) bovine submaxillary mucin (BSM) measured at a frequency of 1 Hz at 25 °C and 37 °C with and without solvent trap. **P*<0.05, ***P*<0.01, ****P*<0.001.

**Table S3. Summary of the macrorheological parameters of sputum from healthy controls and patients with cystic fibrosis.**

|  |  | **Healthy**  **sputum** | | **Cystic Fibrosis sputum** | |
| --- | --- | --- | --- | --- | --- |
|  |  | **mean ± SEM** | ***P* value** | **mean ± SEM** | ***P* value** |
| **G' (Pa)**  **at 1 Hz** | 25 °C without solvent trap | 2.9 ± 0.5 |  | 84.0 ± 42.2 | 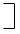  * |
|  | 25 °C with solvent trap | 3.1 ± 0.9 |  | 23.9 ± 10.7 |  |
|  | 37 °C without solvent trap | 3.4 ± 1.0 |  | 4634 ± 3612 |  |
|  | 37 °C with solvent trap | 3.2 ± 0.6 |  | 12.5 ± 3.5 |  |
| **G'' (Pa)**  **at 1 Hz** | 25 °C without solvent trap | 0.9 ± 0.2 |  | 21.7 ± 11.2 | 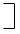  * |
|  | 25 °C with solvent trap | 1.4 ± 0.7 |  | 7.5 ± 3.9 |  |
|  | 37 °C without solvent trap | 1.3 ± 0.4 |  | 1700 ± 1470 |  |
|  | 37 °C with solvent trap | 1.1 ± 0.2 |  | 1.1 ± 0.2 |  |
| **Mesh size (nm) at 1 Hz** | 25 °C without solvent trap | 131.1 ± 15.9 |  | 53.8 ± 6.2 |  |
|  | 25 °C with solvent trap | 136.8 ± 15.6 |  | 73.8 ± 9.7 |  |
|  | 37 °C without solvent trap | 119.5 ± 8.0 |  | 38.0 ± 9.6 |  |
|  | 37 °C with solvent trap | 125.3 ± 10.9 |  | 82.1 ± 8.3 |  |
| **Phase angle (°) at 1 Hz** | 25 °C without solvent trap | 17.4 ± 1.6 |  | 15.0 ± 0.7 |  |
|  | 25 °C with solvent trap | 16.6 ± 1.1 |  | 15.9 ± 0.9 |  |
|  | 37 °C without solvent trap | 21.0 ± 3.0 |  | 16.7 ± 1.3 |  |
|  | 37 °C with solvent trap | 20.3 ± 1.7 |  | 15.5 ± 0.9 |  |

Mean values and standard error of the mean (SEM) of the storage modulus G’ (Pa), loss modulus G’’ (Pa), effective mesh size (nm) and phase angle (°) of sputum from healthy controls (n = 10) and patients with cystic fibrosis (n = 10) measured at a frequency of 1 Hz at 25 °C and 37 °C with and without solvent trap. **P*<0.05.


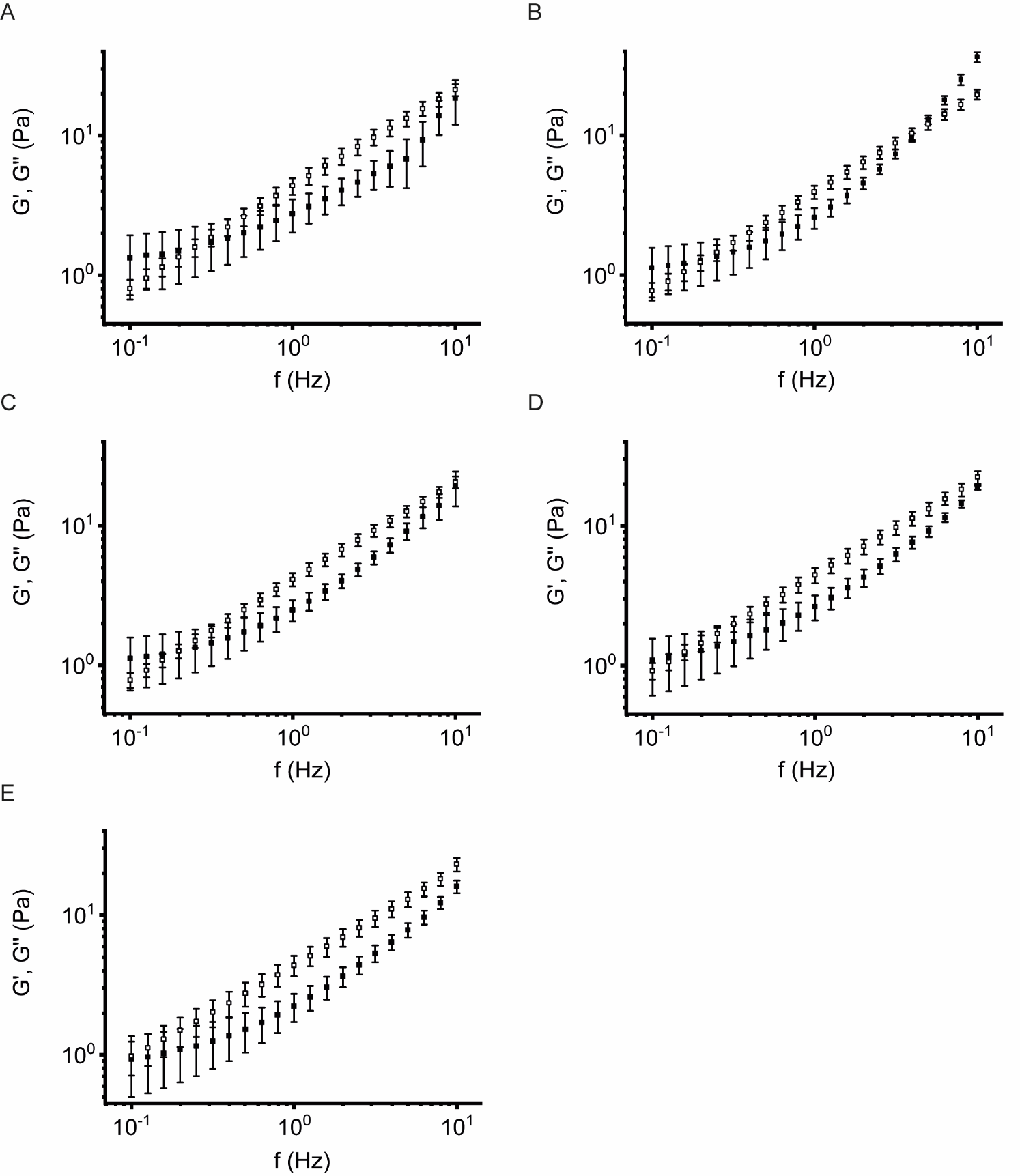


**Figure S1. Deformation effect of different strain amplitudes.** Storage modulus G’ (closed squares) and loss modulus G’’ (open squares) of different strain amplitudes 0.5% **(A)** - 1% **(B)** - 2% **(C)** - 5% **(D)** - 10% **(E)** as function of frequency (Hz) of 10% bovine submaxillary mucin (n = 5) measured at 37 °C with solvent trap.


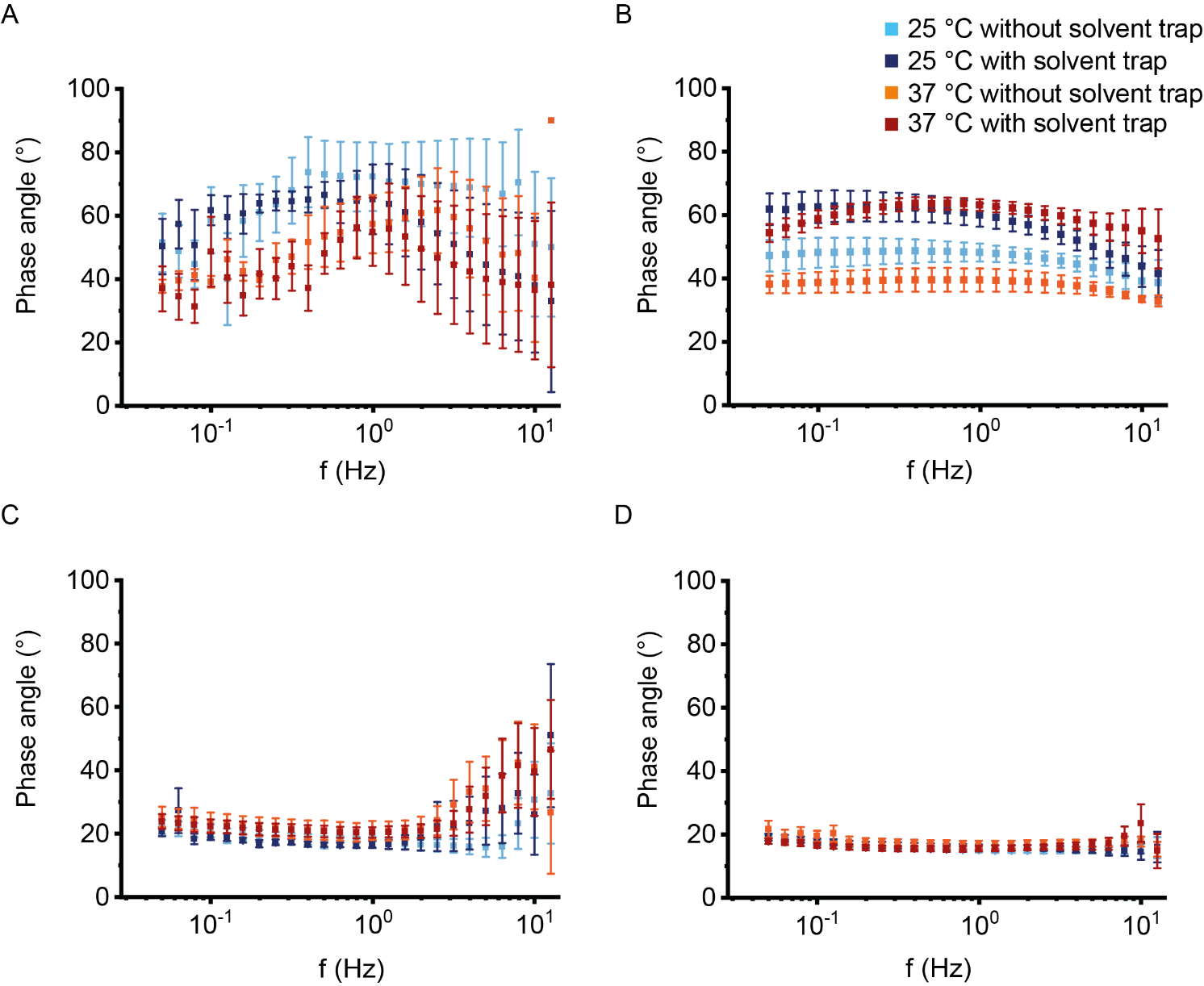


**Figure S2. Phase angle of bovine submaxillary mucin and human sputum.** Phase angle (°) or loss factor of **(A)** 2% (n = 5) and **(B)** 10% (n = 5) bovine submaxillary mucin and of **(C)** sputum from healthy controls (n = 10) and **(D)** patients with cystic fibrosis (n = 10) as function of frequency (Hz). Data are shown as mean ± standard error of the mean (SEM) of measurements at 25 °C and 37 °C with and without solvent trap.

**
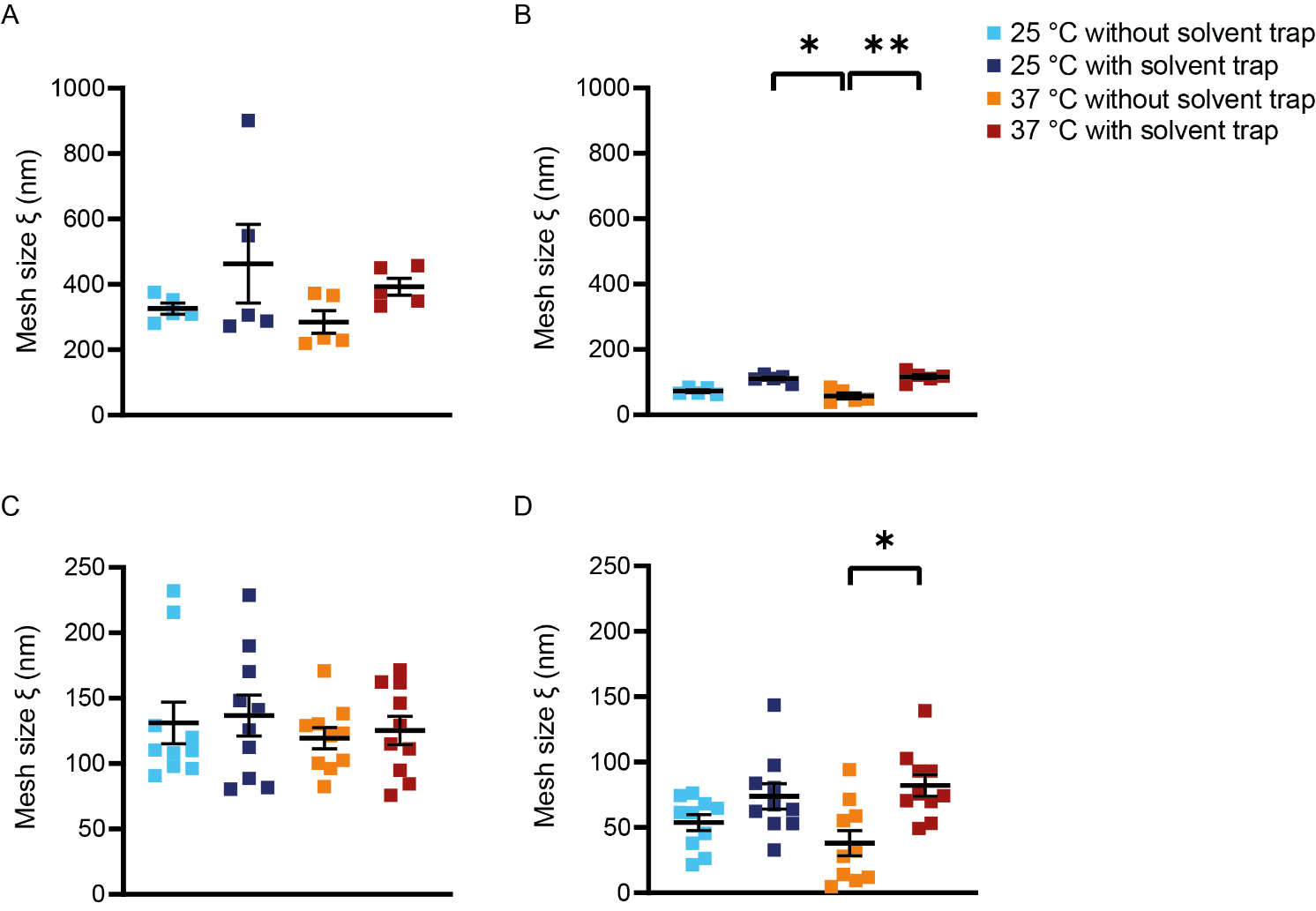
**

**Figure S3.** **Mesh size of bovine submaxillary mucin and human sputum.** Effective mesh size ξ (nm) of **(A)** 2% (n = 5) and **(B)** 10% (n = 5) bovine submaxillary mucin and of sputum from **(C)** healthy controls (n = 10) and **(D)** patients with cystic fibrosis (n = 10). Data are shown as mean ± standard error of the mean (SEM) of measurements at 25 °C and 37 °C with and without solvent trap; **P*<0.05, ***P*<0.01.


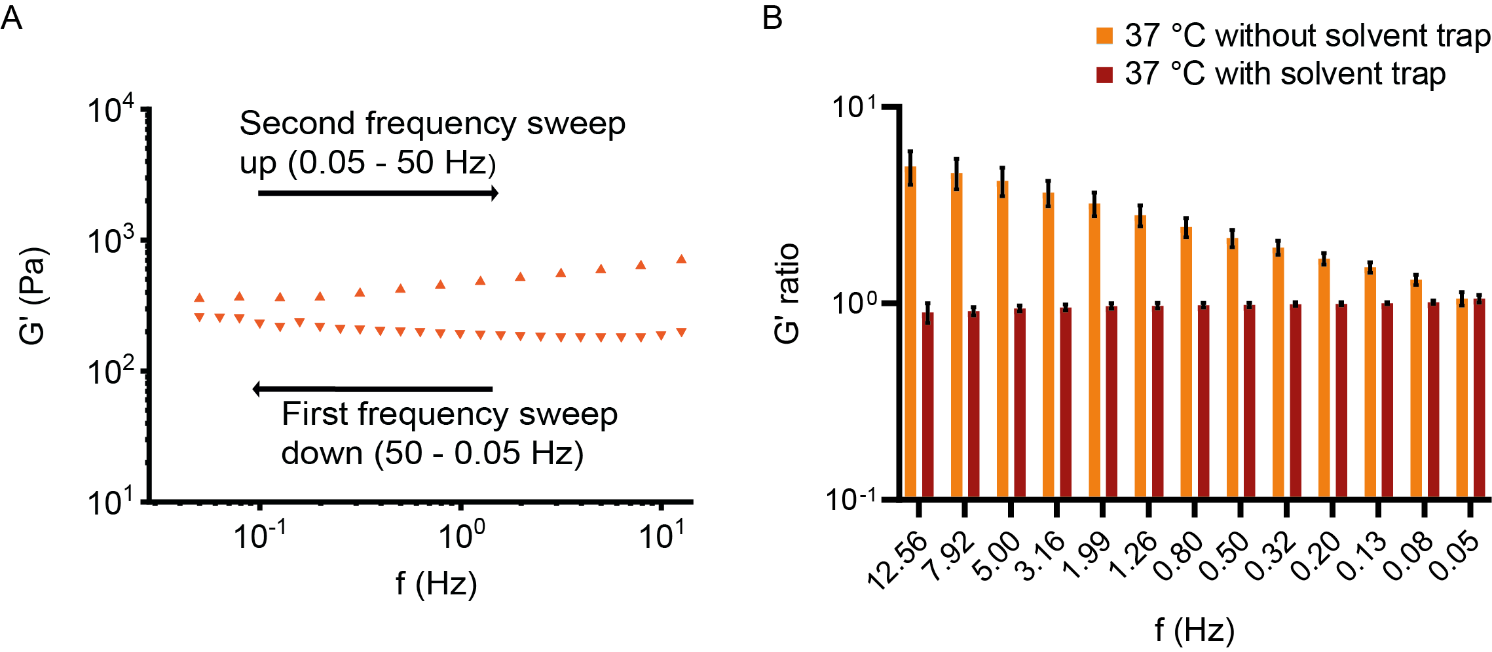


**Figure S4.** **Hysteresis loop of patients with cystic fibrosis.** (**A**) Representative frequency sweep downwards (down-pointing triangle) and upwards (up-pointing triangle) of sputum from a patient with cystic fibrosis at 37 °C without solvent trap. (**B**) Ratio between the storage modulus G’ (Pa) of sputum from patients with cystic fibrosis (n = 10) between the frequency sweep upwards and the frequency sweep downwards at 37 °C without or with solvent trap, respectively. For each frequency mean and standard error of the mean (SEM) is depicted.
